# Formal verification confirms the role of p53 protein in cell fate decision mechanism

**DOI:** 10.1007/s12064-022-00381-x

**Published:** 2022-12-12

**Authors:** Eman Abdelaziz Mahmoud, Mostafa Herajy, Ibrahim E. Ziedan, Hazem I. Shehata

**Affiliations:** 1 Computer and Systems Department, Faculty of Engineering, Zagazig University, Zagazig, Egypt; 2 Mathematics and Computer Science Department, Faculty of Science, Port Said University, Port Said, Egypt; 3 Computer Engineering Department, College of Computing and Information Technology, Shaqra University, Shaqra, Saudi Arabia

**Keywords:** Hybrid modeling, Hybrid Petri nets, Cell fate decision, Model checking, Simulative model checking, PLTL

## Abstract

The bio-cell cycle is controlled by a complex biochemical network of signaling pathways. Modeling such challenging networks accurately is imperative for the understanding of their detailed dynamical behavior. In this paper, we construct, analyze, and verify a hybrid Petri net (HPN) model of a complex biochemical network that captures the role of an important protein (namely p53) in deciding the fate of the cell. We model the behavior of the cell nucleus and cytoplasm as two stochastic and continuous Petri nets, respectively, combined together into a single HPN. We use simulative model checking to verify three different properties that capture the dynamical behavior of p53 protein with respect to the intensity of the ionizing radiation (IR) to which the cell is exposed. For each IR dose, 1000 simulation runs are carried out to verify each property. Our verification results showed that the fluctuations in p53, which relies on IR intensity, are compatible with the findings of the preceding simulation studies that have previously examined the role of p53 in cell fate decision.

## Introduction

The bio-cell cycle is regulated by a biochemical network of signaling pathways (Lee and Cho 2019). If any undesirable external factors interfere with its signaling mechanism, some genes are activated to suppress that danger (Hamada, et al. 2009). Due to the big size and complexity of these biochemical networks, modeling them is intricate, but essential for the understanding of their dynamical behavior (Burke, et al. 2020). The ionizing radiation (IR) stress causes DNA damage in the cell and activation of a tumor suppressor gene called p53 (Mollereau and Ma 2014; Eliaš and Macnamara 2021). p53 regulates the expression of several genes by acting as a transcription factor. These regulated genes induce some cellular responses such as DNA repair, cell cycle arrest, and programmed cell death (apoptosis) (Zhang et al. 2011; Loewer, et al. 2013; Chen 2016). Cell fate decision refers to cellular responses that follow a consistent sequence of biological events starting with p53 gene expression and ending with apoptosis induction. This decision has an impact on tumor cell growth inhibition as well as genetic homeostasis (Kracikova, et al. 2013).

On the one hand, many mathematical models have been developed to investigate how p53 dynamics influence cell cycle arrest and apoptosis induction, see for examples (Hamada, et al. 2009; Zhang et al. 2007; X.-P. Zhang, et al. 2009; Ma, et al. 2005). Zhang et al. in (2009) proposed a four-module integrative model including generation/repair of Double-Strand Breaks (DSB) induced by IR and cell fate decision along with others. According to this study, the possibility of changes in DSB generation caused cell-to-cell variability in cell fate. Similarly, K. Iwamoto (Iwamoto, et al. 2014) studied the fluctuations in p53 pulses generation and developed a mathematical model of DSB generation/repair system, p53 signaling network, and apoptosis induction pathway to better understand the major factors that influence the fate of the cell.

On the other hand, the use of graphical modeling languages, such as Petri nets (David and Alla 2010; Gilbert and Heiner 2013; Gilbert and Heiner 2006; Matsuno et al. 2011; Liu et al. 2020) for modeling complex networks simplifies the interpretations of their dynamical behavior. They also help in identifying their missing kinetic parameters. As an example, the authors in (Machado, et al. 2012) demonstrated a model reduction technique for large-scale metabolic networks using the Petri net framework and applied that to the E. Coli central carbon metabolism model as a case study. Hybrid Petri nets (HPN) (David and Alla 2010) are used in (Matsuno, et al. 2000) to model gene regulatory networks after being hierarchically arranged like their representation in biochemistry. Generalized hybrid Petri nets (Herajy and Heiner 2012) are used in (Herajy et al. 2013) to investigate certain biological phenomena that are related to cell cycle and its complex biochemical networks such as cell division, cell replication, and cell growth. More examples of modeling biological systems using HPN can be found in (Herajy et al. 2013; Herajy et al. 2017; Ismail, et al. 2020; Herajy et al. 2018a, b).

Furthermore, model checking (Clarke et al. 2018) is an important tool that has been extensively used for verifying many types of systems. Model checking is used in (Donaldson and Gilbert 2008) for estimating the parameter values in biochemical models. The behavior of the biochemical species is represented using a variant of the probabilistic linear temporal logic (PLTL).

A model of signaling transduction reaction network that is related to an intriguing protein (named HMGB1) in eukaryotic cells is presented in (Gong, et al. 2010). A statistical model checking technique (Younes and Simmons 2006) is applied to verify some properties about that model. Simulative model checking is used in (C. Rohr 2013) to verify unbounded large stochastic models of biochemical reaction networks.

In this paper, we utilize HPN to model one of the significant biochemical networks that encompasses a mixture of stochastic and continuous behavior and includes hundreds of proteins interacting with each other in the cell. We model the sequence of biological events which is triggered when the cell gets exposed to an external factor (such as IR) and causes a DNA damage. These biological events start with p53 gene expression in the nucleus until it reaches apoptosis induction in the Cytoplasm. These events decide the cell fate and suppress the growth of the tumor cells (Kracikova, et al. 2013).

Our main contributions in this paper can be summarized as follows:

- Building a quantitative but a graphical hybrid model for the biochemical network that controls the cell fate by the help of hybrid Petri nets. The model can be easily executed to produce simulation results that are used afterward during the verification phase.
- Capturing both the stochastic and deterministic effects of the target network as shown by the dynamical behavior of the model in an intuitive way that can be easily understood by biologists.
- Formally, verifying the model important properties, written in PLTL, using simulative model checking.

The rest of the paper is organized as follows: Section 2 covers related work of the paper. Section 3 gives a brief introduction of simulative model checking, PLTL, Petri nets, transformation from chemical reactions to Petri nets, as well as background information about the biological network controlling the cell fate decision. Section 4 describes our HPN model of cell fate decision and the simulative model checking technique used for verifying the HPN model. Section 5 discusses the simulation and verification results produced by the hybrid simulation engine and MC2 model checker. Finally, Section 6 highlights the conclusions of this research.

## Related work

In Iwamoto et al. (2014), a mathematical model of the system of DSB generation and repair, the signaling network of p53, and the pathway of apoptosis induction is constructed to investigate the major factors that determine the probability of cell survival upon exposure to different irradiation intensities. In their study, reactions that take place inside nucleus are modeled as stochastic processes, while reactions that happen in the cytoplasm are modeled as deterministic processes. Both the intra-nuclear and cytoplasmic reactions are simulated simultaneously in a hybrid simulation environment. Cells in that model are exposed to IR doses of 0, 0.3, 2.5, 6.0 Gy. Their simulation results show many sustained oscillations of p53, ATM, Mdm2, and Wip1. The authors have also asserted that the number of p53 pulses varies according to IR intensity at the single-cell level. Moreover, at the cell population level, the model generates damped oscillations of p53, and the IR intensity affects the first p53 pulses amplitudes. Statistical analysis of their simulation results illustrates that the generation of several p53 pulses is a precondition for inducing apoptosis. Also, the stochasticity of the intranuclear biochemical reactions is responsible for the final cell fate decision accompanied by the DNA damage. However, no formal verifications of the model results have been performed and the constructed model is a plain system of ODEs with time jumps to execute stochastic events.

In (Machado, et al. 2012), the authors proposed conjunctive and disjunctive methods to model reduction in metabolic networks based on Petri nets. The conjunctive approach transforms a subnetwork into a single macro reaction, but it is restricted to a specific flux distribution, while the disjunctive approach replaces the deleted subnetwork with macro reactions for all possible pathways with no constraints on the flux distribution, hence not restricting the space of the steady-state solutions. If experimental data is available, then using parameter estimation and kinetics reading can transform the reduced model into a dynamical model. The proposed methods are applied to model the E. coli dynamical central carbon metabolism model.

The authors in (Matsuno, et al. 2000) introduced a method to represent gene regulatory networks using hybrid functional Petri nets. This approach is applied to the genetic switch mechanism of λ phage to express transcription and translation of genetic information of an operon with two genes. Afterward, they have been extensively applied the idea of functional Petri nets to many biological case studies, e.g., see (Matsuno et al. 2011). In (Herajy et al. 2013), generalized hybrid Petri nets are employed to model the eukaryotic cell cycle by utilizing a self-modifying arc weight to be able to represent cell division in a cell cycle model. This idea has been considerably extended to colored hybrid Petri nets (Herajy et al. 2018a, b) permitting the modeling of yeast cell cycles based on multisite phosphorylation. Snoopy – a Petri net tool to construct, animate and simulate different classes of type of Petri net classes (Heiner et al. 2012) are used to implement such models.

Similarly, model checking techniques (Souri, et al. 2019; C. Rohr 2013; Mardare, et al. 2005; Heath, et al. 2006) can be adapted to validate deterministic biochemical pathway models and describe their behavior using Probabilistic Temporal Logics (PTL) (Donaldson and Gilbert 2008; C. Rohr 2013). PTL is used in (Donaldson and Gilbert 2008) to describe behaviors of biochemical pathways on both deterministic and stochastic levels. This approach estimates the distance between a model’s behavior and its desired behavior. In addition, many similar studies are focused on using a variety of model checking techniques to validate biochemical pathways (Gong, et al. 2010; C. Rohr 2013; Heath, et al. 2006; Calder, et al. 2006; Cho, et al. 2003; Napione, et al. 2009). In (Gong, et al. 2010), a model of signaling transduction reaction network induced by HMGB1 is developed. BioNetGen rules are used to express the model. Ordinary Differential Equations (ODEs) and the Stochastic Simulation Algorithm (SSA) (Gillespie 2007) are used to simulate this model. In addition, statistical model checking (David, et al. 2015) can automatically verify the model outcome regarding its known experimental results.

Furthermore, model checking techniques have also been used extensively to validate popular stochastic biochemical pathway models. For instance, in (C. Rohr 2013), an algorithm for unbounded time limit model checking and a steady-state operator for probabilistic linear-time temporal logic are presented. The proposed algorithm is based on stochastic simulation. Its simulation runtime depends on the model size and rate functions rather than the state-space size. The main drawback of this method is its accuracy since the number of required simulations runs grows exponentially with its expected accuracy (Calder, et al. 2006). This approach is applied to a common chemical pathway such as ERK pathway repressed by RKIP (Cho, et al. 2003) and angiogenetic process models (Napione, et al. 2009).

Our paper continues in the same direction as this related work by first constructing a graphical model of DSB generation and repair, the signaling network of p53, and the pathway of apoptosis induction based on the one previously constructed in (Iwamoto, et al. 2014). Moreover, HPN are selected as a modeling tool for these scenarios. Afterward, the model is validated using simulative model checking to ensure that the model result is consistent with known biological facts.

## Modeling and verification tools: petri nets and model checking

In this section, we briefly present the tools used in this paper to construct a HPN model of cell fate decision as well model verification tools to validate the model properties.

### Petri nets

Petri nets, e.g., see (Heiner et al. 2008) are a graphical formalism for modeling concurrent systems. They can support qualitative as well as quantitative (stochastic and continuous processes) modeling and therefore they have been extensively applied for studying and analyzing biological systems (Machado, et al. 2012; Matsuno, et al. 2000). Using Petri nets, it would be possible to represent concurrent and parallel processes in a single model, while enjoying the graphical structure that captures both temporal and spatial aspects of a system in a comprehensive way.

A Petri net is represented by a directed, finite, bipartite graph, typically without isolated nodes (Heiner et al. 2008). The four main components of standard Petri nets are: places, transitions, arcs and tokens. In the one hand, places are passive nodes, indicated by circles, which can represent e.g., species in a biochemical network and they can carry tokens. Tokens are indicated by a number within a place that may represent concentration level (continuous) or number of molecules (discrete) of species. Places also have initial markings representing the initial states of a model. On the other hand, transitions are active nodes, represented by square, they may refer to biochemical reactions. Moreover, directed arcs (also called edges) are inactive elements, indicated by arrows, they carry arc weights that set the number of tokens consumed or produced by a transition. Arc weight may represent the stoichiometry of biochemical reactions. In addition to this static structure, Petri nets distinguish themselves from other modeling languages by their dynamical execution. That is, a transition may consume tokens from its preplaces and produce tokens to its post places according to arc weights via a process called transition firing. Each class of Petri nets have firing rules that control the firing of transitions and the time of firing due to their rate functions.

In what follow, we briefly present the formal definitions of Petri net classes that are of interest in this paper, namely stochastic Petri nets, continuous Petri nets and hybrid Petri nets. A complete and detailed presentation of all Petri net classes can be found in (David and Alla 2010).

#### Formal definitions of petri nets

##### Stochastic petri nets

A stochastic Petri net (SPN) (Heiner et al. 2008) is a quintuple $(\mathrm{P},\mathrm{ T},\mathrm{ f},\mathrm{ v}, {m}_{0})$, where:

- P, T are two finite, non-empty, disjoint sets representing the discrete places and stochastic transitions, respectively.
- $\mathrm{f}: ((\mathrm{P}\times \mathrm{T})\cup (\mathrm{T}\times \mathrm{P}))\to {\mathbb{N}}$ defines a set of directed arcs, weighted by non-negative integer values.
- $\mathrm{v}:\mathrm{ T}\to {\mathbb{R}}_{+}$ maps every transition to the corresponding rate of the exponential distribution of the delay of the transition firing.
- ${m}_{0}: \mathrm{P}\to {\mathbb{N}}$ represents the initial marking of discrete places (non-negative integer values).

A transition gets enabled if its pre-places are sufficiently marked. Before firing of an enabled transition $t \in T$, a waiting time has to elapse. The waiting time is an exponentially distributed random variable ${X}_{t}\in \left(0,\infty \right).$

SPNs are usually used to model the application scenarios that exhibit randomness during their execution. For example, in the biochemical reaction networks, reactions that involve species with low number of molecules need to be stochastically simulated in order to correctly capture their semantics (Ashraf, et al. 2018; Liu et al. 2016)

##### Continuous petri nets

A continuous Petri net (CPN) (David and Alla 2010; Heiner et al. 2008) is a quintuple $(\mathrm{P},\mathrm{ T},\mathrm{ f},\mathrm{ v}, {m}_{0})$, where:

- P, T are two finite, non-empty, disjoint sets representing the continuous places and transitions, respectively.
- $\mathrm{f}: ((\mathrm{P}\times \mathrm{T})\cup (\mathrm{T}\times \mathrm{P}))\to {\mathbb{R}}_{+}$ describes the set of directed arcs, weighted by non-negative real values.
- $\mathrm{v}:\mathrm{ T}\to {\mathbb{R}}_{+}$ defines for each transition $t$ a marking-dependent continuous firing rate.
- ${m}_{0}: \mathrm{P}\to {\mathbb{R}}_{+}$ is the initial marking of the continuous places (nonnegative real values).

CPNs can be considered as another way of performing deterministic simulation. They are of paramount importance when simulating models with abundance number of molecules where stochastic simulation is not of a big help because of the long simulation time. Please note that there are different interpretations of the underlying semantics of CPN (Herajy and Heiner 2018a, b). In this paper, we are more interested in the bio-semantics of CPN where the execution of transitions is closely related to the deterministic simulation and the undelaying semantics of a system of ODEs.

##### Hybrid petri nets

A hybrid Petri net (HPN) (David and Alla 2001; David and Alla 2010) is a combinations of stochastic and continuous Petri nets. An HPN can be defined as a 6-tuple $(\mathrm{P},\mathrm{ T},\mathrm{ h},\mathrm{ Pre},\mathrm{ Post},{M}_{0})$, where:

- $P, T$ are two finite, non-empty, disjoint sets where $P$ is the set of places and $T$ is the set of transitions.
- $h: P\cup T\to \{D,C\}$ is called the “hybrid function”. It differentiates between discrete and continuous nodes.
- $Pre: P\times T\to {\mathbb{N}} \mathrm{or }{\mathbb{R}}_{+}$(discrete or continuous) is the input incidence mapping. $Pre({P}_{x},{T}_{y})$ specifies the weight of the arc that goes from the place ${P}_{x}$ to the transition ${T}_{y}$.
- $Post: P\times T\to {\mathbb{N}} \mathrm{or }{\mathbb{R}}_{+}$ (discrete or continuous) is the output incidence mapping. $Post({P}_{x},{T}_{y})$ specifies the weight of the arc that goes from transition ${T}_{y}$ to place ${P}_{x}$.
- ${M}_{0}: P\to {\mathbb{N}} \mathrm{or }{\mathbb{R}}_{+}$ (discrete or continuous) is the initial markings of the places.

In addition to the standard HPN introduced in (David and Alla 2010), different extensions of HPNs have been defined in (Matsuno et al. 2011) and (Herajy and Heiner 2012) to better fit the modeling of biological systems. Table 1 shows the different elements of hybrid Petri nets (Herajy et al. 2013) used to represent our model. More details about deterministic, scheduled, and immediate transitions can be found in Heiner, et al. 2009.

**Table 1:** Graphical representation of hybrid Petri net elements used in our model

Elements	Places	Representation	Transitions	representation	Edges	representation
Element representation	Discrete place		Discrete or Stochastic		Standard	
Element description	Holds a non-negative integer number expressing number of molecules of species		Fires with a random delay distributed exponentially		Connects a place to a transition or vice versa and can carry discrete or continuous weights. permits its connected transition to be enabled if pre-place marking exceeds its arc weight	
Element representation	Continuous place		Continuous		Modifier	
Element description	Holds a non-negative real number expressing species’ concentration		Fires continuously		Connects only a place to a transition and has no arc weight	

A hybrid Petri net can contain discrete and continuous places as well as discrete and continuous transitions. Places and transitions are connected together via arcs which can carry weights

#### Modeling rules

In building our model, we have employed the following rules systematically based on the suggested ideas in (Gilbert and Heiner 2013; Gilbert and Heiner 2006):

1. All types of chemical reactions in the system (such as synthesis, degradation, phosphorylation, activation, association and dissociation) are represented as transitions in the model.
2. All species, irradiation, genes, MRN, proteins, complex proteins and input signals are represented as places in the model.
3. The relationships between places and transitions are captured by arcs whose weights define the stoichiometry of the chemical reactions.
4. Species with low number of molecules are modeled via discrete place, while those which exhibit high level of molecules are represented by continuous transition.

To connect stochastic and deterministic subnetworks of a hybrid model, certain rules should be followed, e.g., see (Herajy and Heiner 2012; Herajy et al. 2013).

Table 2 shows an example of the connection between nucleus (modeled by a stochastic Petri net) into cytoplasm (modeled by a continuous Petri net) subnetworks. In this example, a stochastic transition is used to model the transportation, while a discrete place is used as a pre-pace to this transition and a continuous place is used a post-place to represent the reaction output.

**Table 2:** Hybrid Petri net representation of species transportation between stochastic and continuous parts

Biochemical reaction	Hybrid Petri net representation	Propensity function
Transport A from nucleus (${A}_{n}$) into Cytoplasm (${A}_{c}$) with rate k		${k* A}_{n}$

In what follow, we present how the different kinds of our model reactions can be represented as stochastic, continuous and hybrid Petri nets.

##### Transforming biochemical reactions into a stochastic petri net

As the timing behavior of biochemical reactions usually exhibits randomness, stochastic Petri nets are the right choice to represent them. Table 3 shows the representation of association and dissociation of chemical proteins and complexes as well as other reaction types represented as stochastic Petri nets.

**Table 3:** Transforming chemical reactions into stochastic Petri nets

Biochemical reaction	Stochastic Petri net representation	Propensity function
Auto synthesis reaction: protein A is produced with synthesis rate s		$s$
Synthesis reaction: protein B is induced by A with rate s		$s*A$
Degradation reaction: A is degraded with degradation rate d		$d*A$
Dissociation reaction: complex A_P is dissociated into two species of A (phosphorylated A)$A\_P\to 2\mathrm{A}$		$k*A\_P$
Association reaction: protein A binds to B protein resulting in the complex A_B with binding rate f $$A+B\to A\_B$$		$$f*A*B$$
Dissociation reaction: complex A_B dissolve into Species A and B with dissociation rate r $A\_B\to A+B$		$r*A\_B$

The Petri nets in this table illustrate common biochemical reactions including auto synthesis, synthesis, degradation, disassociation and association reactions

##### Transforming deterministic reactions into continuous Petri net

Table 4 summarized the set of rules used to model the reactions that express the interactions of species (deterministic processes in cytoplasm) to continuous Petri nets. When a CPN is simulated, each place will have an ordinary differential equation (ODE) that represents its dynamics. The corresponding ODEs are generated by the help of known kinetic laws (e.g., Mass action or Michaelis–Menten kinetics), see (Herajy and Heiner 2018a, b) for more details.

**Table 4:** Continuous Petri net representation of ODE's

Reaction	ODE	Continuous Petri net representation
Auto degradation reaction	$\frac{dA}{dt}= -d*A$	
Synthesis reaction	$\frac{dB}{dt}= s*A$	
Association and dissociation reactions	$$\frac{dA}{dt}= -f*A*B+r*A\_B$$ $$\frac{dB}{dt}= -f*A*B+r*A\_B$$	

Each ODE variable is represented by a continuous place. Common rates are represented by transitions. The final ODE is generated by considering the difference between the rate of pre and post transitions of each place

### Simulative model checking

Model checking (Clarke et al. 2018) is an automated technique for verifying temporal properties about models of concurrent systems. The properties are typically expressed in temporal logics, which are formal languages that support specific sets of propositional and temporal operators. The model of the system is explicitly represented as a graph or symbolically represented as a Boolean formula and exhaustively analyzed to prove/disprove the property. Such an analytical approach is sound (i.e., handles all possible scenarios) and static (i.e., does not need to run the model) yet suffers from scalability problems (i.e., the state-space explosion problem).

An alternative to such an analytical approach of model checking is to execute (or simulate) the model many times and check whether (or how often) the property is satisfied in these simulation runs (Donaldson and Gilbert 2008). This technique is referred to as simulative model checking. Checking the property can take place either during the simulation (i.e., on-the-fly) or afterward (i.e., offline) (C. Rohr 2013). Simulative model checking has the potential to handle relatively complex systems however, unlike analytical model checking, the accuracy (and/or the soundness) of its results usually depends on the number of simulation runs (C. Rohr 2013).

### PLTL (probabilistic linear temporal logic)

Temporal logics are a formal means to specify temporal properties about concurrent systems. Since they were first introduced (Łos 1947) (around 1947), several variants of temporal logics have been developed. The most well-known (and used) ones are the linear temporal logic (LTL) (Pnueli 1977; Vardi 1996) and the computation tree logic (CTL) (Clarke and Emerson 1981; Boucheneb and Hadjidj 2006). Each of these logics has its own pros and cons. For instance, LTL is relatively more intuitive and easier to use while CTL is less complex to model-check (Bloem et al. 1999).

Many temporal logics have been extended to be more quantitative and able to specify properties about probabilistic systems. Examples of these probabilistic temporal logic extensions are probabilistic CTL (PCTL) (Hansson and Jonsson 1994), continuous stochastic logic (CSL) (Aziz, et al. 1996) and probabilistic LTL (PLTL) (Ognjanovic 2006; C. Rohr 2013).

In this paper, we chose to specify our properties in PLTL because it results in path formulas which are more suitable to simulative model checking which handles individual runs of the model. The syntax of PLTL can be represented in Backus normal form (BNF) form as follows:

$$\psi : = {\mathcal{P}}_{ = ?} \left[ \varphi \right] | {\mathcal{P}}_{\bowtie}{{x}} \left[ \varphi \right]$$

where $\bowtie \in \left\{<,>,\le ,\ge ,=,\ne \right\}$, $x\in [0, 1]$, and $\varphi$ is an LTL formula with the following syntax:

$$\varphi : = true \left| { false } \right| \alpha \left| { \beta \bowtie \beta \left| { \neg \varphi } \right| \varphi \wedge \varphi \left| { \varphi \vee \varphi } \right| X \varphi \left| { G \varphi } \right| F \varphi } \right| \varphi U \varphi$$

where $\alpha$ is an atomic proposition (i.e., Boolean variable), $\beta$ is a number, numerical variable, or arithmetic expression. The symbols “${\text{X}}$”, “${\text{G}}$”, “${\text{F}}$”, and “${\text{U}}$” represent the well-known LTL temporal operators “next”, “globally”, “eventually”, and “until” respectively.

As can be deduced from its syntax, PLTL extends LTL syntax with the probability operator $\mathcal{P}$ which can be used in one of two modes. When it is used with the question mark, ${\mathcal{P}}_{=?}[\varphi ]$ denotes the probability that the LTL formula $\varphi$ is satisfied. When it is used with a relational operator $\bowtie$ and a probability value $x$, ${\mathcal{P}}_{ {\bowtie} x} \left[ \varphi \right]$ is satisfied if and only if the probability of satisfying $\varphi$ ${\bowtie}x$.

## Materials and methods

### Model construction

**Fig. 1:**
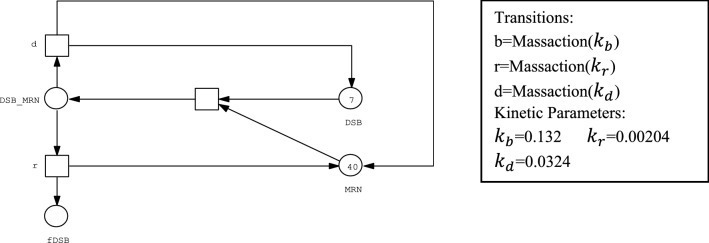
Representation of DSB generation/repair system as a stochastic Petri net: DSB, MRN and fDSB are represented by discrete places while their operations are modelled by stochastic transitions

**Fig. 2:**
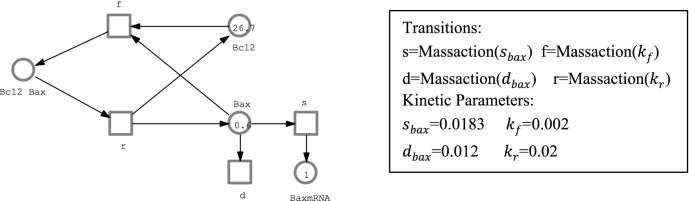
Representation of composition of Bax in Cytoplasm as a continuous Petri net

In this section, we present our hybrid Petri net model of cell fate decision mechanism. The model is focused on the generation and repair system of DSB, the signaling network of p53, and the apoptosis induction mechanism presented in (Iwamoto, et al. 2014). It includes 29 discrete places, 52 stochastic transitions representing reactions in the nucleus and 45 continuous places, 69 continuous transitions controlling the biochemical reactions in the Cytoplasm. Figure 1 is a representation of DSB generation/repair system using stochastic Petri net, while Fig. 2 is a representation of composition of Bax in cytoplasm represented as continuous Petri nets.

**Fig. 3:**
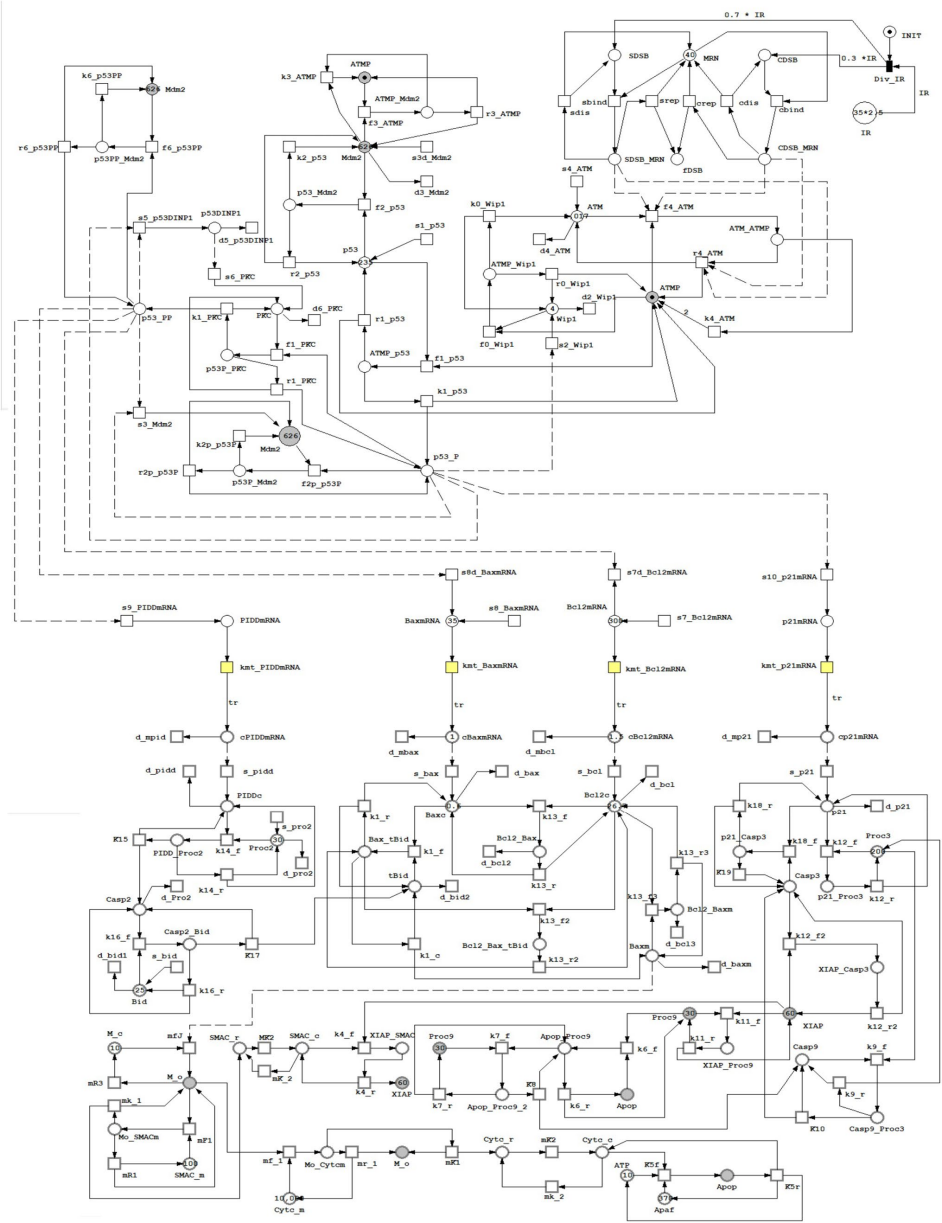
The hybrid Petri net model of cell fate decision mechanism. Places represent biochemical species, transitions represent reactions. Please note that places with same names are logical places which are used to simplify network connections

Figure 3 shows the complete hybrid model. The hybrid Petri net model introduced in this section is based on the previous mathematical model by K. Iwamoto (Iwamoto, et al. 2014). The same kinetic Parameters, initial conditions of nuclear and cytoplasmic species, biochemical reactions in the nucleus and ordinary differential equations for Cytoplasmic reactions of the previous stated model (Iwamoto, et al. 2014) are also used in our model. In our proposed model, DSB generation/repair system and p53 Signaling network (in Nucleus) are stochastically modeled using their propensities whereas the intrinsic mechanism for apoptosis induction (in Cytoplasm) is deterministically modeled using continuous Petri nets.

The input to the network model DSB is produced due to DNA damage. It is calculated from Poisson random distribution with a mean value 35 * IR where IR values are 0.3, 2.5, or 6 Gy. DSB is separated into simple Double Strand Breaks (sDSB) and complex Double Strand Breaks (cDSB) such that 70% of DSB is modeled as sDSB and 30% is modeled as cDSB.

In what follow, we discuss in more details the different model components.

Our experiment flow is automated using a script written in Java and consists of three steps:

1. Modeling the biochemical network of interest as a hybrid Petri net in Snoopy (Herajy, Liu and Rohr, et al. 2017).
2. Generating CSV traces of the hybrid Petri net model using the command-line Petri net simulator Spike (Chodak and Heiner 2018), Spike also internally uses the same simulation engine (Herajy and Heiner 2012; Herajy and Heiner 2018a, b) as Snoopy.
3. Eventually, the Petri net model is verified via Monte Carlo Model Checker (MC2) (Donaldson 2020).

#### Intra-nuclear biochemical reactions

The intra-nuclear part of the model consists of DSB system generation and repair as well as p53 signaling network. The upper part of the model in Fig. 3 represents reactions that take place in nucleus while the lower part represents reactions that happen in cytoplasm.

After the system is subjected to an IR dose, DNA is damaged and DSB is induced. In our model irradiation dose is denoted by the discrete place IR and the marking of this place represents the amount of IR. The effect of the IR is modeled by the firing of the immediate transition Div_IR. The used IR that induced DSBs is based on the mathematical model introduced by Ma et al. in (Ma, et al. 2005) that deduced the total number of DSB created using a Poisson distribution with a mean of 35x, where the value of x is 0, 0.3, 2.5, or 6 Gray. sDSB (as place SDSB), and cDSB (as place CDSB) are the two types of DSBs, with 70% modeled as sDSB and 30% as cDSB. The ratio of sDSB to cDSB is modelled by the initial marking of the two places sDSB and cDSB. The ratio of sDSB to cDSB is modelled by the arc wights connecting the transition Div_IR to place SDSB and place CDSB, respectively. Indeed, marking-dependent arc weights introduced in (Machado, et al. 2012) proved to be useful to model such semantics.

DSB-MRN complex (places CDSB_MRN and SDSB_MRN) induces auto phosphorylation of ATM (produces ATM-P). ATMP activates Ser15 of p53 (p53p) and degrades Mdm2 that causes the degradation of the three formulas of p53 (p53, p53-P, and p53-PP). Mdm2, p53DINP1, and Wip1 levels in the nucleus are increased by p53-P. p53-P also stimulates the production of p21 mRNA. p53-PP promotes the synthesis of Bax_mRNA and PIDD_mRNA while inhibiting the Bcl-2_mRNA synthesis (Smeenk, et al. 2011). Wip1 deactivates ATMP (forming ATM) resulting in a negative feedback loop between p53 and ATM (Shreeram, et al. 2006). Negative feedback loops are built between p53 and Mdm2, as well as between ATM and p53.

#### Apoptosis induction pathway (deterministic processes in the cytoplasm)

The resulting p21_ mRNA, PIDD_ mRNA, Bax_ mRNA, Bcl2_ mRNA is synthesized in the nucleus by interfering of p53p, p53pp and quickly transported from nucleus to cytoplasm. These transported mRNAs induce the synthesis of proteins p21, PIDD, Bax, and Bcl2 in the Cytoplasm. In an ATP-dependent mechanism, Apoptotic protease activating factor-1 (Apaf-1), Cytc, and ATP interact in the cytoplasm to generate an Apoptosome (Apop) (Rodriguez and Lazebnik 1999).

In the apoptosis induction pathway, Cytc activated the caspase cascade (Taylor et al. 2008). Casp9 and Casp3 are activated when an apoptosome (Apop) binds to its associated procaspase (Proc9 or Proc3) (Rodriguez and Lazebnik 1999). Binding of the XIAP (XLinked Inhibitor Apoptosis) protein inhibits both Casp-3 and Proc-9 (Datta et al. 2000) but, SMAC constrains XIAP protein and regulates Casp-3 generation (Srinivasula, et al. 2000).

Bcl-2_c inhibits apoptotic induction by binding to both cytoplasmic and mitochondrial Bax. P21_c binds to Proc-3 in the cytoplasm and prevents Casp-9 from splitting it. (Suzuki, et al. 1998). On the other hand, Casp-3 allows the degradation of p21_c, causing the cell cycle arrest forced termination (Zhang et al. 1999).

In our proposed model, Casp3 is an indicator of apoptosis induction. More specifically, high levels of Casp3 indicate cell death, whereas low levels of Casp3 indicate cell survival.

#### Hybrid Petri net representation of mRNAs transferred from nucleus to cytoplasm

**Fig. 4:**
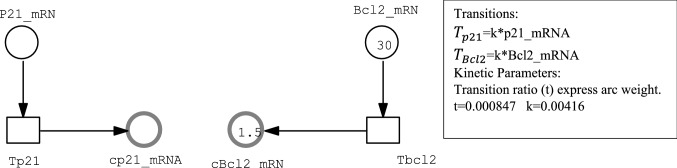
Hybrid Petri net representation of a transformation part from nucleus to cytoplasm

Figure 4 illustrates how the transformation between the nucleus and cytoplasm is modelled. In this figure, stochastic transitions represent the transformation from stochastic part to continuous one. The connected arcs between stochastic transitions and continuous places carry weights equals the transformation ratio.

Transformation ratio represents the transformation from the number of molecules (N) to concentration (nM). Hybrid representation required the translation of the species (p21_mRNA, Bax_mRNA, Bcl2_mRNA, and PIDD_mRNA) translocated from the nucleus to the cytoplasm. It is calculated using the equation: $C=\frac{N}{{N}_{A} {V}_{c}}$ where C represents concentration, N represents the number of molecules, N represents the Avogadro constant, and V is the cytoplasmic volume estimated as 1.96 PL by Ciliberto (Ciliberto et al. 2005), then the used trans ratio = 0.00847.

### Simulating the model

The biochemical system explained in Sect.  4.1 is modeled using Snoopy (Heiner et al. 2012) as a hybrid Petri net by following the method explained in Sect. 3.1.2. After that, the model is exported to an ANDL file (which is more suitable format for simulation using the command-line simulator Spike) (Chodak 2019). To perform the simulation, for each IR dose (0.3, 2.5, 6 Gy), a script is used to generate a thousand variants of the model in which the input DSB is assigned random values according to a Poisson distribution whose mean value is 35*IR, and the DSB components are defined such that sDSB = 0.7*DSB and cDSB = 0.3*DSB. All model variants are then simulated using Spike and the results are saved as CSV files for offline analysis and verification.

### The model verification

Simulative offline model checking is used in this paper to check the behavior of the proposed model. The tool selected for this purpose is Monte Carlo Model Checker (MC2) (Donaldson 2020) which is a model checker to check PLTL properties on the data extracted from a quantitative simulator. Our goal is to calculate the probabilities of whether p53 would have 0, 1 or more pulses. To do so, we define three PLTL properties that describe these three cases respectively.

#### Property P1:

What is the probability that p53 has no pulses?

$${\mathcal{P}}_{=}\left[{\text{G}} \, \left( p53<{T}_{L}\right)\right]$$

The p53 has no pulses when p53 is generally lower than a specific low threshold value ${T}_{L}$ and never exceeds it.

#### Property P2:

What is the probability that p53 has only one pulse (p53 rises then fall and reaches steady state at low level)?

To eliminate the effect of noise on the signal on simulation results, we use upper threshold ${T}_{U}$ and lower threshold ${T}_{M}$ values to identify the pulse as a pulse signal starts with lower threshold and then eventually increase until it exceeds an upper threshold ${T}_{U}$.

$${\mathcal{P}}_{ = } \left[ {\left( {p53 < T_{M} } \right) \wedge F \left( {p53 > T_{U} } \right) \wedge F \left( {G \left( {p53 < T_{M} } \right)} \right) \wedge G \left( {\left( {p53 > T_{U} } \right) \to \left( {\left( {p53 > T_{M} } \right) U \left( {G \left( { p53 < T_{U} } \right)} \right)} \right)} \right)} \right]$$

#### Property P3:

What is the probability that p53 has 2 or more pulses?

It is achieved when p53 has neither one pulse nor any pulses.

$${\mathcal{P}}_{ = } \left[ {\neg \left( {\left( {p53 < T_{M} } \right) \wedge F \left( {p53 > T_{U} } \right) \wedge F \left( {G \left( {p53 < T_{M} } \right)} \right) \wedge G \left( {\left( {p53 > T_{U} } \right) \to \left( {\left( {p53 > T_{M} } \right) U \left( {G \left( { p53 < T_{U} } \right)} \right)} \right)} \right)} \right) \wedge \neg \left( {G \left( { p53 < T_{L} } \right)} \right)} \right]$$

**Fig. 5:**
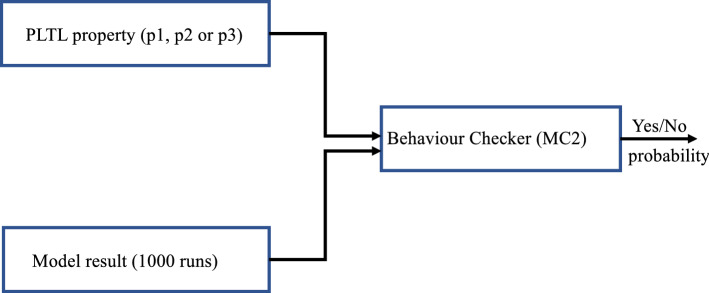
Model checking the properties in our hybrid Petri net model

To verify each property (P1, P2, or P3), we run the Monte Carlo Model Checker (MC2) 1000 times for each IR dose (0.3, 2.5, 6 Gy). During the runs as shown in Fig. 5, we feed MC2 with the CSV files generated by Spike (as explained in Sect.  4.2) one by one and then calculate the average of the outcome probabilities for each property and IR dose.

## Results and discussion

In this section, we present the paper results and examine the outcome of the model verification.

### Simulation results

Before looking at the formal validation of the model, we explore the simulation output. This is achieved by inspecting the IR effect on p53 and other intranuclear species dynamics on individual and multiple cells as well as intrinsic apoptosis induction.

#### IR effect on p53 and other intranuclear species dynamics

##### Individual cells

**Fig. 6:**
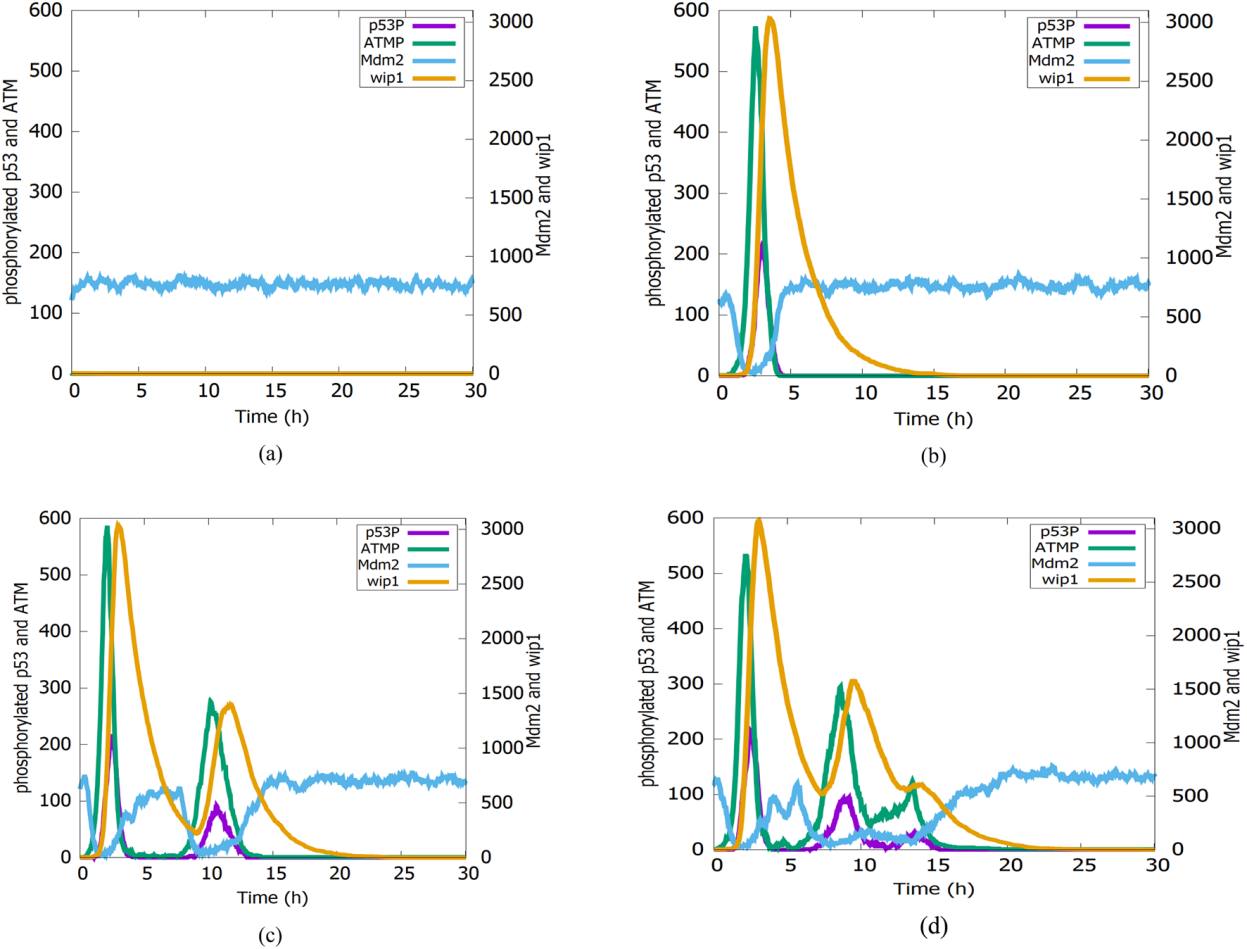
Simulation results of nuclear species (phosphorylated p53, Mdm2, ATM-P and Wip1) following an IR-dose of 2.5 Gy in four individual cells with: **a** No, **b** one, **c** two and **d** three p53 pulses. Respective species represents the summation of complexes

Figure 6 shows the simulation results of phosphorylated p53, Mdm2, Wip1, and ATMP in four different cells subjected to an IR dose of 2.5 Gy. By observing the simulation results, we note the differences in the number of the p53 pulses that cause changes in p53 dynamics. The previous research (Lahav, et al. 2004), which is based on experiments performed on a population of MCF7 cells subjected to IR, deduced that the number of p53 pulse varies among the cells. Also, in (Batchelor, et al. 2008) the authors demonstrated that p53, ATMP, and Mdm2 components in the p53 signaling network exhibited oscillation. Moreover, the mathematical model of that network constructed by Iwamoto (Iwamoto, et al. 2014) confirmed the same results. The simulation results of our Petri net model (shown in Fig. 6) are in the same line with all these biological findings (Lahav, et al. 2004; Batchelor, et al. 2008) and also the mathematical modeling simulation results of Iwamoto (Iwamoto, et al. 2014).

##### Multiple cells (overall)

**Fig. 7:**
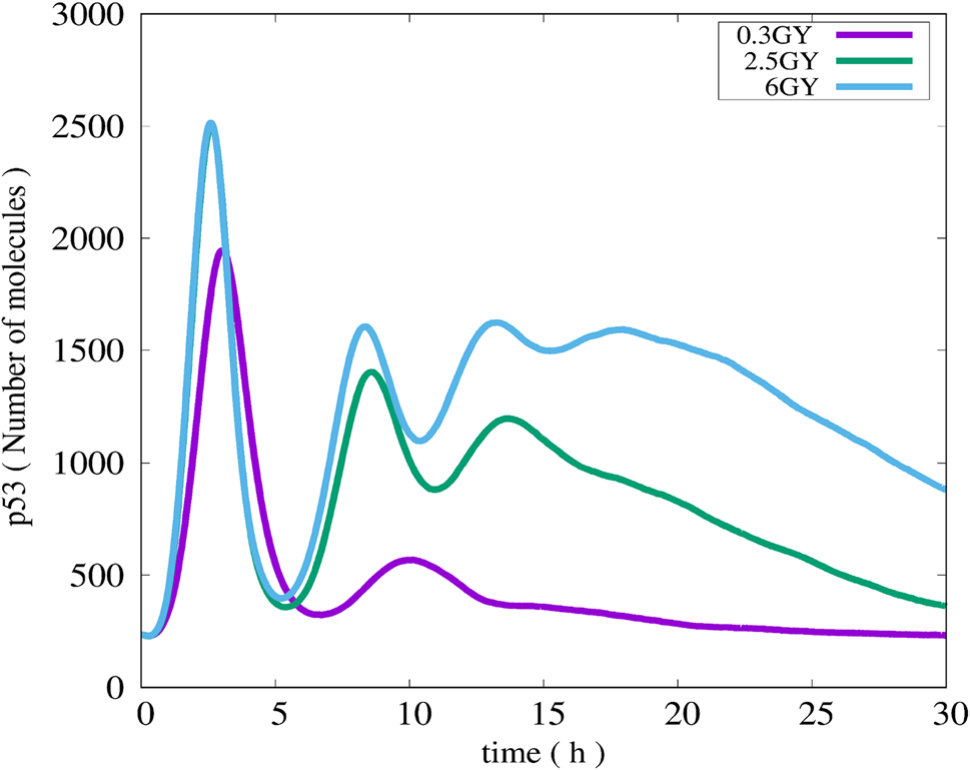
Simulation results of total p53 (Summation of p53 complexes) for populations of 1000 runs subjected to IR doses of 0.3, 2.5, and 6 Gy

As shown in Fig. 7, it is noticed that at the cell population level (populations of 1000 runs), the number of p53 pulses increases as the IR dose increases. Furthermore, the amplitude of the first pulse increases as the IR dose increases. These results are in compliance with a report of Bar-or et al. (Bar-or, et al. 2000) which deduced that both p53 and Mdm2 shows decreasing oscillation in a population of NIH3T3 cells exposed to IR.

Damped oscillation is observed in the simulation results of populations of 1000 runs for our hybrid Petri net model. Such damped oscillation implies that the corresponding biological system reaches a relax-stable state. Our simulation results are in a good agreement with a report of Iwamoto (Iwamoto, et al. 2014) that deduced the occurring of damped oscillation of p53 at the cell population level and a relaxed-stable state of the system in that case.

#### Intrinsic apoptosis induction

**Fig. 8:**
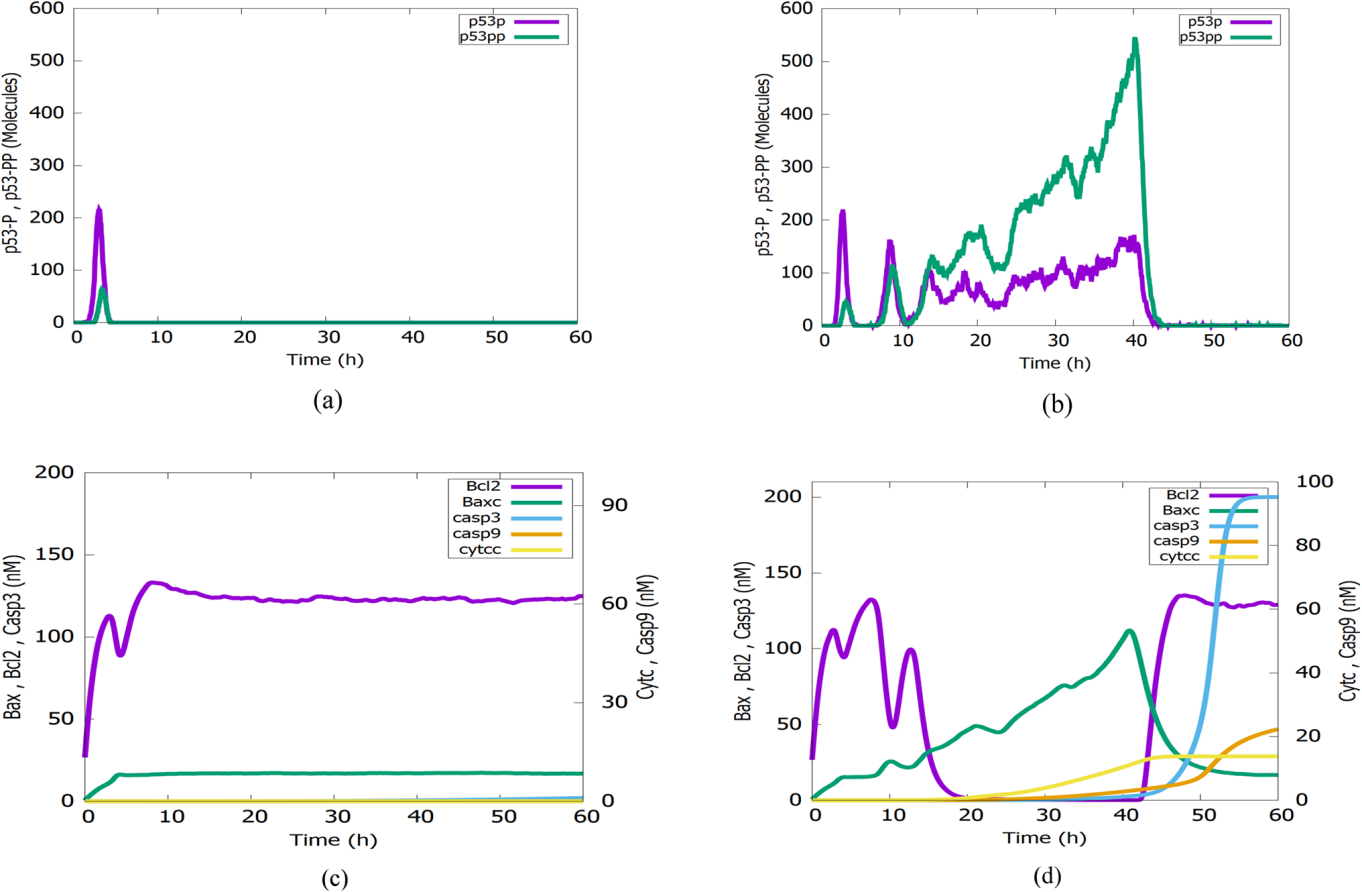
Simulation results of cytoplasmic species Bax, Bcl2, Cytc, Casp9, Casp3 subject to an IR dose of 2.5 GY for 2 individual cells and the effect of p53 on them (**a**, **c**) Generation of one pulse of p53pp doesn’t activate Casp3 (**b**, **d**) Generation of successive p53 pulses causes activation of Casp3

Fluctuations in the p53 dynamics in the nucleus impact the apoptosis induction pathway in the cytoplasm as shown in Fig. 8. In Fig. 8a, the generation of one pulse of p53 causes low levels of Bax concentration and high levels of BCl2 concentration in the cytoplasm as shown in Fig. 8c. These results cause non-activation of Casp3 and non-induction of apoptosis.

In Fig. 8b, there is a generation of multiple p53pp pulses and over increasing p53pp pulses after the second pulse. It causes low and high levels of Bcl2 and Bax sequentially as in Fig. 8d. They induce Casp3 activation and apoptosis induction.

The simulated results in Fig. 8 are in good agreement with both the biological findings presented in (Pastorino, et al. 1998), and the mathematical model of the report stated in (Iwamoto, et al. 2014). Both studies conclude that the changes in p53pp lead to a rapid increase of Bax and activation of Casp3 which consequently induces the apoptosis of the cell.

### Verification of the proposed model

Since our model is relatively large and has a mixture of stochastic and continuous processes, we use offline simulative model checking (by MC2 tool) to verify the properties introduced in Sect.  4.3. The three threshold values are carefully selected as follows:

${T}_{L}$ =920, ${T}_{M}$=1200, ${T}_{U}$=1800 for 2.5 and 6 Gy. The verification results for the three properties P1, P2, P3 are illustrated in Table 5 below.

**Table 5:** Verification results of P1, P2, and P3

IR dose	Property 1(no p53 pulses)	Property 2 (one pulse of p53)	Property 3 (two or more p53 pulses)
0.3	0.082	0.651	0.267
2.5	0.007	0.432	0.561
6	0.001	0.372	0.626

The outcome probabilities result indicates that when the IR increases, the number of generated p53 pulses also increases. The simulation runs are carried out using a machine running a windows10—64-bit operating system, intel core i7 processor—quad core—2.7GHZ, and 16 GB RAM. Interval splitting used in our simulation is 2000. Runtime is shown in Table 6.

**Table 6:** Summary of runtime

Runs	Individual 1000 runs	Populations 1000 run	Checking the properties for 1000 runs for each Gray
Time	Average 5 min to generate each run Average 3 days and 12 h for the complete generation of the 1000 runs for 0.3 Gy	Time duration to generate 1000 runs: for 6 Gy is 70 min, 20 secs For 0.3 Gy is 64 min, 8 secs	Average 5 min to check each property

## Conclusions

In this paper, a unified model of DSB generation and repair system, the signaling network of p53, and apoptosis induction pathway is developed using hybrid Petri nets. Snoopy, Spike. Afterward, MC2 tools are then employed to validate the HPN model.

The constructed model assumes IR doses of 0.3, 2.5 and 6 Gy and shows the cell responses to those three stress levels. These stresses induce DNA damage (DSB) as expected from biological observations. The obtained simulation results of the hybrid Petri net model showed that

- At the single-cell level (individual runs): there are several persistent oscillations of p53, Mdm2, ATM, Wip1, and fluctuations in the number of p53 pulses based on IR intensity.
- At the cell population level (population of 1000 runs): there are damped oscillations of p53 which indicate that the system reaches a stable state. IR dose intensity also affects the amplitude of the first p53 Pulse.

These simulated results are consistent with various biological discoveries in MCF7 and NIH3T3 cells, indicating that our suggested model is biologically sound. The analysis of our simulation results demonstrates that the fluctuations in intra-nuclear biochemical reaction processes affect the cell survival chances. Simulative model checking is used to verify some Properties in our Petri net model. The results of these properties confirm that our model is biologically sound as when IR intensity increases, fluctuations in the number of p53 pulses also increase.

In future, we aim to study the possibilities of applying modeling/verification techniques used in this paper to other biochemical networks. We aim also to consider using other statistical modeling/verification tools such as PRISM (Kwiatkowska et al. 2011) and Plasma-Lab (Legay et al. 2016).

Moreover, the model presented in this paper consists of a substantial number of places and transitions. While the model dimension is still under control when modelled as a low-level Petri net, colored Petri nets will render the model much more readable. Thus, an extension of our hybrid model into a color hybrid Petri net can also be part of future work.

## Data Availability

The datasets used, the constructed model and materials are available from the corresponding author upon request.

## Abbreviations

DSB: Double strand breaks
ATM: Ataxia telangiectasia mutated
Chk2: Checkpoint kinase 2
Mdm2: Mouse double minute 2 homolog
Wip1: Wild-type p53-induced phosphatase
XIAP: X linked inhibitor apoptosis
sDSB: Simple double strand breaks
cDSB: Complex double strand breaks
Apaf1: Apoptotic protease activating factor-1
Proc: Procaspase
Apop: Apoptosome
IR: Ionizing radiation
casp: Caspase
PLTL: Probabilistic linear time temporal logic
HMGB1: High mobility group box 1
